# Rare Incidental Finding of an Interventricular Membranous Septal Aneurysm in an Adult Presenting for Aortic Valve Replacement

**DOI:** 10.7759/cureus.80519

**Published:** 2025-03-13

**Authors:** Danielle Sawka, Amy Arthur, Andrew Maslow, Shyamal R Asher

**Affiliations:** 1 Anesthesiology, The Warren Alpert Medical School of Brown University, Providence, USA; 2 Anesthesiology, Rhode Island Hospital, Brown University, Providence, USA

**Keywords:** adult congenital heart disease, aortic stenosis (as), bicuspid aortic valve disease, interventricular membranous septal aneurysm, transesophageal echocardiography (tee)

## Abstract

Interventricular membranous septal aneurysm (IVMSA) is a rare cardiac anomaly that typically coexists with other cardiac malformations. Recognition of IVMSAs is important for risk mitigation of potentially serious complications such as right ventricular outflow obstruction and proper characterization of the lesion is critical for planning of surgical repair. Literature reports of this condition are sparse and appear limited to case reports. Bicuspid aortic valve (BAV) is a common congenital cardiac malformation tending to present later in life with symptomatic stenosis and is frequently associated with other defects. We report an unusual case of an elderly woman with a BAV undergoing aortic valve replacement who was incidentally diagnosed with an IVMSA. This case report describes intraoperative transesophageal echocardiogram (TEE) characterization of the IVMSA used to help plan the surgical procedure. TEE accurately visualized the IVMSA’s exact anatomic location and impact on cardiac function which led to the decision to surgically repair the IVMSA with an autologous patch. This report also includes a comparative discussion of clinical presentations, diagnostic approaches, and management strategies in previously published IVMSA case reports and considerations for operative management.

## Introduction

The incidence of congenital cardiac anomalies is approximately 0.9%. Bicuspid aortic valve (BAV) and ventricular septal defect (VSD) are the two most common abnormalities, with respective incidences in the general population of 1.3% and 0.3% [1]. BAVs may occur in tandem with other cardiovascular defects including ascending aortopathy, aortic arch coarctation, and a myxomatous mitral valve [2,3]. In 70% of cases, BAV coexists with a second congenital cardiac defect [4]. Up to 80% of patients with BAV will require surgery during their lifetime for resultant aortic valve stenosis and/or insufficiency [5,6]. Therefore, a comprehensive cardiac assessment is necessary to formulate an appropriate surgical plan.

An interventricular membranous septal aneurysm (IVMSA) is a rare congenital anomaly with no accurate incidence characterized by an outpouching of the membranous interventricular septal wall. While IVMSAs are reported in about 0.3% of all congenital cardiac lesions, they may occur in 19% of patients with ventricular septal defects [7]. IVMSA development appears related to the relative pressure gradient on the left and right side of the thin membranous septum. Vulnerabilities in the membranous septum may be idiopathic or acquired from spontaneous VSD closure, consequences of other massive congenital cardiac anomalies distorting septal anatomy, or prior infection or trauma [8]. Typically, IVMSA are found incidentally and are asymptomatic. They may, however, predispose patients to arrythmias, infection, right ventricular outflow obstruction, embolism from a thrombus formed in the aneurysmal sac, or even rupture [7,8]. The potential for worsening of our patient’s dyspnea from unrepaired IVMSA right ventricular outflow obstruction was a key factor in deciding on surgical repair.

While the coexistence of a BAV and VSD is known to occur [9], the combination of a BAV and IVMSA with or without a prior VSD is exceedingly rare in the literature with descriptions limited to case reports [7]. The following case report describes a patient with bicuspid aortic valve stenosis, a dilated aorta, and an incidental finding of an IVMSA. The unique transesophageal echocardiographic findings of IVMSAs are described, including the literature on IVMSAs, and considerations for anesthetic and surgical management are discussed.

## Case presentation

Informed consent was obtained from the patient for publication of this report including accompanying images. A 72-year-old female with a past medical history significant for coronary artery disease, bicuspid aortic valve on transthoracic echocardiogram (TTE) and right bundle branch block presented with three years of worsening dyspnea on exertion. She was found to have severe aortic stenosis (aortic valve area (AVA) 0.6 cm2) and mild coronary artery disease (CAD) without angina (50% left anterior descending artery (LAD) and 30% right coronary artery (RCA)). These lesions were deemed non-obstructive and managed medically. The patient was referred for a surgical aortic valve replacement (SAVR).

**Figure 1:**
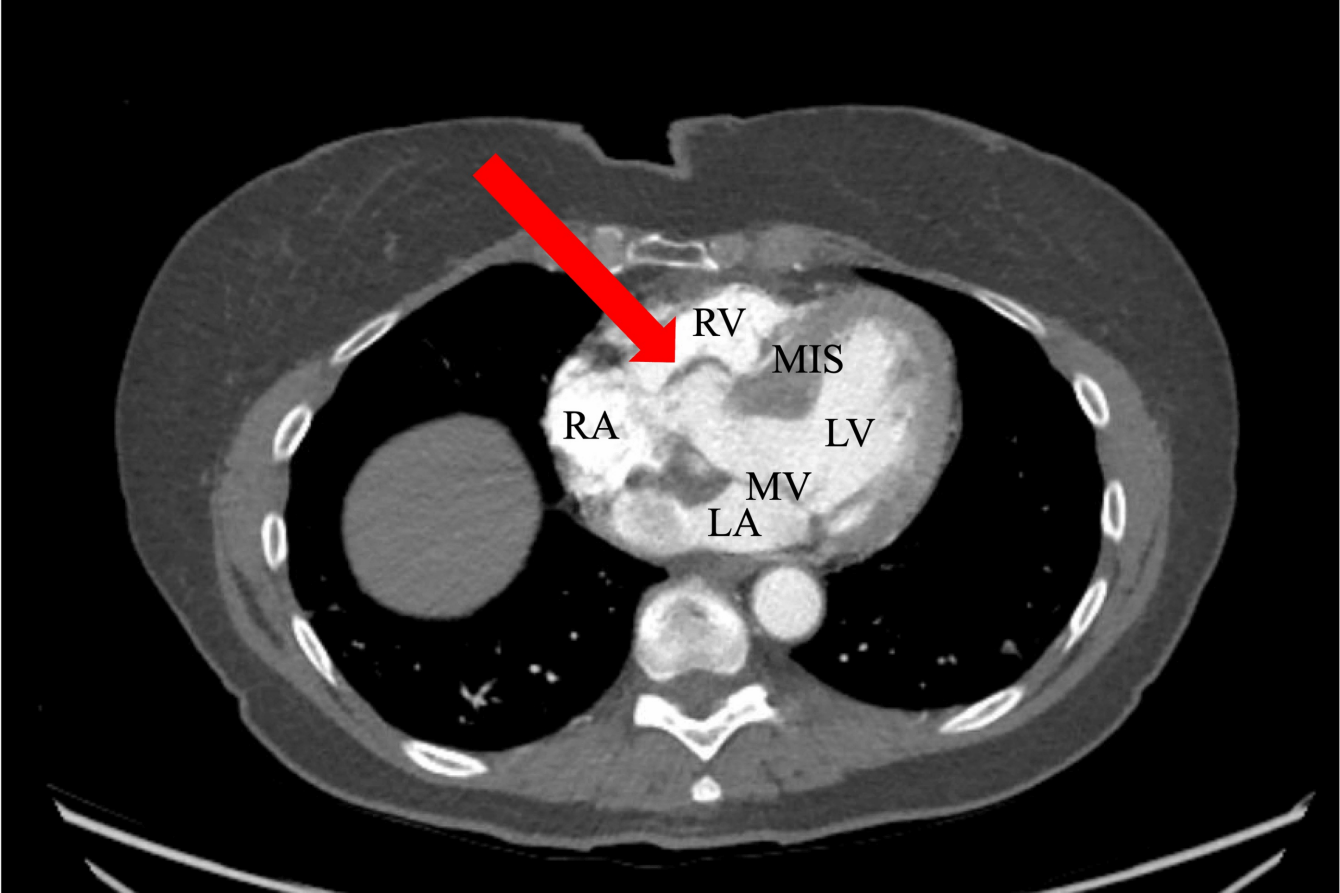
Computed tomography (CT) angiography of chest depicting the interventricular membranous septal aneurysm (arrow), right atrium (RA), right ventricle (RV), left atrium (LA), mitral valve (MV), left ventricle (LV), and muscular interventricular septum (MIS).

Preoperative computed tomography (CT) angiography of the chest confirmed severe aortic valve calcification with an Agatston score of 2887. The Agatston score, or coronary artery calcium measurement, is a risk marker of cardiovascular morbidity and mortality with scores over 1000 considered very high [10]. This imaging also demonstrated dilated aortic root (diameter 41 mm) and an aneurysm of the membranous interventricular septum (2.4 cm) as seen in Figure 1.

**Figure 2:**
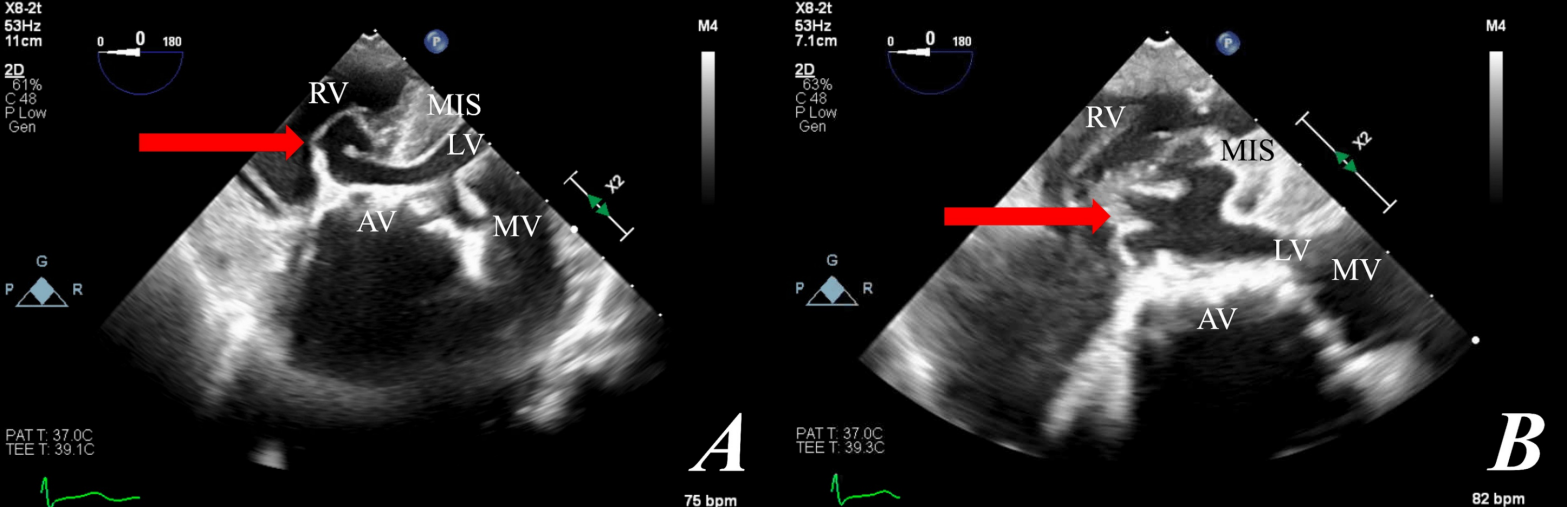
A) zoomed out B) zoomed in. A deep trans gastric view showing the interventricular membranous septal aneurysm (arrow), aortic valve (AV), mitral valve (MV), right ventricle (RV), left ventricle (LV), and muscular interventricular septum (MIS).

After multidisciplinary discussion, the decision was made to further characterize this finding intraoperatively using transesophageal echocardiogram (TEE). Unlike CT angiography, TEE with Doppler enabled visualization of the aneurysm’s movement in real time and diagnostic certainty regarding the presence or absence of interventricular flow. Intraoperatively, TEE visualized an intact membranous ventricular septal wall with billowing of the septum into the right ventricular outflow tract, anomalous motion, and no interventricular flow or shunting, thus confirming the diagnosis of an IVMSA (Figure 2A, 2B). The aortic valve annulus diameter was measured at 2.3 cm. Doppler ultrasound revealed an aortic valve mean gradient of 42 mmHg, with no evidence of insufficiency. The ascending aorta was 4 mm in diameter. Otherwise, TEE was unremarkable and absent of other pathologies.

After transverse aortotomy, the diseased aortic valve was excised and the IVMSA was inspected. The IVMSA was described as fibrous tissue with trabeculae, showed no communication with surrounding structures and the excess tissue billowed into the right ventricular outflow tract.

Through the aortotomy and prior to aortic valve placement, the IVMSA was repaired with an autologous pericardium patch sewn into the fibrous rim of the defect. Following this, a 25 mm Inspiris Resilia bovine valve (Edwards Lifesciences, Irvine, CA, USA) was placed and the aortotomy closed. No intervention was performed on the aorta. Post-bypass TEE revealed left ventricular ejection fraction of 70% and no billowing of the repaired membranous defect. A mild intravalvular leak from the bioprosthetic aortic valve commissures was noted and considered consistent with normal valve function. The bioprosthetic aortic valve mean gradient was 7 mmHg. Post-surgery, the patient was transported to the cardiothoracic intensive care unit in stable condition with an unremarkable postoperative course resulting in discharge on postoperative day five.

## Discussion

Congenital cardiac anomalies have an approximate incidence of 0.9%, with a BAV the most common type [1]. In contrast, IVMSAs are exceedingly rare, representing about 0.2% of congenital cardiac defects [7]. IVMSAs can be found in patients who underwent surgical repair as infants for emergent cardiac anomalies, particularly transposition of the great arteries [8]. We present a therefore unusual case of an unexpected IVMSA in a 72-year-old woman with no prior cardiac surgeries who presented for symptomatic aortic stenosis secondary to a bicuspid aortic valve.

The interventricular septum includes four parts: the muscular part, the membranous part, the inlet, and the infundibular part. Closest to the heart’s apex is the thick muscular septum. Midway along the septum lies the weaker membranous septum which lacks myocardial tissue; it is the upper and posterior part of the interventricular septum and separates the aortic vestibule from the lower part of the right atrium and the upper part of the ventricle. The inlet is trabeculated and lies over the inferoposterior portion of the septum while the infundibular septum is between the right and left ventricular tracts [9]. The pathophysiology of IVMSA development is related to relative weakness of the membranous septum which is then subject to the high-pressure differential between the left and right ventricles. The septal weakness often appears to be acquired postnatally [8]. Potential complications include rupture, arrythmia, thromboembolism secondary to hemostasis, endocarditis, right ventricular dysfunction, or outflow tract obstruction leading to subpulmonic stenosis [8,9]. The present patient was noted to have a right bundle branch block (RBBB) on several prior electrocardiograms and a one-year history of intermittent palpitations, which plausibly arose from her IVMSA. There is a known connection between IVMSAs and conduction disturbances, apparently due to stretching of the nerve fibers coursing through the dilated membranous septum [8,11]. Echocardiographic assessment did not report right ventricular dysfunction, right ventricular outflow obstruction or any significant right ventricular outflow tract (RVOT) gradient despite billowing of the IVMSA into the right ventricular outflow tract.

Although it is unclear if the IVMSA meaningfully contributed to the patient’s dyspnea in addition to the dyspnea caused by her severe aortic stenosis, the IVMSA was considered as a cause of RBBB and her recent onset of intermittent palpitations one year prior to surgery. The expansion of an unrepaired IVMSA over time causing further dyspnea was a relevant possibility, and therefore due to the combination of these factors, the IVMSA was repaired.

A summary of salient published case reports on IVMSA within the last 15 years is presented in Table 1. There is a wide range of presentations and treatments. Our 72-year-old patient was older than all other patients described, where patient ages range from three to 69 years old [7,12-19]. Unlike our patient, many patients had prior cardiac surgery [13,17,18]. A relatively common finding across these IVMSA case reports is a right ventricular outflow billowing and obstruction, the former of which visually appeared to be present on our patient’s imaging [7,12,15,16,18]. Most patients from Table 1 had an initial TTE which prompted additional imaging such as a TEE. TTE, TEE, and CT angiography are the three main imaging modalities to diagnose and assess the significance of IVMSA. Given the overall rarity of IVMSA images in the literature, the standard use of TEE for IVMSA assessment in this case is nevertheless notable for adding to the body of visual presentations and enhancing visual understanding. Management of IVMSA ranges from no intervention to conservative medical management to, as in our patient, surgical repair. Of the nine patient cases in Table 1, less than half of the IVMSAs were surgically repaired. RVOT obstruction appears to be a common clinical feature among patients whose IVMSA was surgically repaired [12,13,16,18]. Neither treatments nor IVMSA dimensions were described in several of the available case reports making it more difficult to infer patterns between IVMSA characteristics and management decisions. Among recent IVMSA literature, the presented case therefore appears to be the first such combination of an elderly patient without prior cardiac surgery, three different imaging modalities, and successful surgical repair.

**Table 1:** Summary of Recent Interventricular Membranous Septal Aneurysm (IVMSA) Case Reports in the Literature TTE = transthoracic echocardiogram, TEE = transesophageal echocardiogram, CTA = CT angiography, VSD = ventricular septal defect, NYHA = New York Heart Association, RVOT = right ventricular outflow tract, BAV = bicuspid aortic valve

Study	Case Details	Imaging	Repair
Bakr et al. (2020) [12]	Five-year-old male with dyspnea since birth was found to have IVMSA with large VSD causing RVOT obstruction and stenosis.	TTE	Successful surgical resection of aneurysmal tissue via right atriotomy with patch repair
Yarrabolu et al. (2015) [13]	Three-year-old male with dextrocardia and congenitally corrected transposition of the great arteries found to have VSD, IVMSA, and severe pulmonary stenosis.	TTE, CTA	Successful surgical repair with VSD closure and aneurysm resection
Sharma et al. (2017) [14]	69-year-old female with chronic history of supraventricular tachycardia and intermittent palpitations on metoprolol presented with worsening of symptoms. A new 2.2 x 1.5 x 1.7 cm IVMSA was found incidentally.	TTE, CTA	Conservative management with metoprolol and aspirin with six-month follow-up
Naidu et al. (2012) [7]	1) 18-year-old female athlete with bradycardia found to have an incidental 1.2 x 0.9 cm IVMSA caudal to right coronary sinus; otherwise asymptomatic; 2) 60-year-old male NYHA Class III found to have severe aortic valve stenosis and 2.2 cm x 1.7 cm x 1.6 cm IVMSA without associated VSD projecting into the right ventricle.	1) TTE, CTA, TEE; 2) TEE, CTA	1) No intervention; 2) Surgical replacement of aortic valve via aortotomy; no surgical intervention of IVMSA.
Privitera et al. (2017) [15]	69-year-old female with dyspnea NYHA Class II with 9 mm VSD and IVMSA causing dynamic RVOT obstruction.	TTE, TEE	None described.
Chen et al. (2011) [16]	55-year-old female diagnosed in childhood with BAV and small VSD who presented with new-onset dyspnea found to have IVMSA with RVOT obstruction.	TTE, TEE	Successful surgical resection of IMVSA, VSD closure, tricuspid valve annuloplasty.
Razzouk et al. (2013) [17]	25-year-old male with congenitally corrected transposition of great arteries as a child who presented with acute hemoptysis, found to have abnormal positioning of atrioventricular valves, found to have IVMSA causing subpulmonic stenosis and left ventricular outflow tract obstruction.	CTA, TTE, TEE	None mentioned
Velicki et al. (2018) [18]	60-year-old female with past surgical closure of atrial septum defect and pulmonary valve commissurotomy who presented with dyspnea found to have a 3 x 2 cm IVMSA causing RVOT obstruction.	TTE, TEE	Surgical transaortic plication of IVMSA and aortic valve replacement

Perioperative care of a patient with IVMSA might include anticoagulation or prophylactic antibiotics. Anticoagulation therapy is indicated in the presence of a visualized thrombus and prophylactic antibiotics are indicated if the patient has a cyanotic congenital heart disease [19]. Given the sparse literature on IVMSAs, there appear to be no formal standardized guidelines. Determination of billowing and the presence of RVOT narrowing or obstruction are important preoperative determinations to guide fluid management and expectant hemodynamic issues that might be related. While no adverse effects from the IVMSA arose in this patient intraoperatively, increased vigilance by the anesthesiologist was necessary to ensure her hemodynamic stability. If the IVMSA causes RVOT obstruction and subsequently subpulmonic stenosis, this is an especially critical perioperative risk that may not be present in other cardiac surgeries.

## Conclusions

IVMSA is a rare cardiac defect characterized in the literature chiefly through case reports. IVMSAs can predispose patients to thrombogenic and arrhythmogenic events, or subpulmonic stenosis secondary to the protrusion of the aneurysm into the right ventricular outflow tract. This report adds to the literature by further describing this condition in an atypical presentation and offering excellent visualization of the defect on transesophageal echocardiogram, one of several potential diagnostic modalities. Clinical significance of IVMSA in our patient and in general is reviewed to help guide perioperative care by anesthesiologists. A summary of current IVMSA case reports, the imaging modalities employed, and interventions are detailed for comparative review.
